# Treating Senescence like Cancer: Novel Perspectives in Senotherapy of Chronic Diseases

**DOI:** 10.3390/ijms21217984

**Published:** 2020-10-27

**Authors:** Alessia Mongelli, Sandra Atlante, Veronica Barbi, Tiziana Bachetti, Fabio Martelli, Antonella Farsetti, Carlo Gaetano

**Affiliations:** 1 Laboratorio di Epigenetica, Istituti Clinici Scientifici Maugeri IRCCS, Via Maugeri 4, 27100 Pavia, Italy; alessia.mongelli@icsmaugeri.it (A.M.); sandra.atlante@icsmaugeri.it (S.A.); veronica.barbi@icsmaugeri.it (V.B.); 2 Direzione Scientifica, Istituti Clinici Scientifici Maugeri IRCCS, Via Maugeri 4, 27100 Pavia, Italy; tiziana.bachetti@icsmaugeri.it; 3 Laboratorio di Cardiologia Molecolare, Policlinico San Donato IRCCS, San Donato Milanese, 20097 Milano; Italy, fabio.martelli@grupposandonato.it; 4 Institute for Systems Analysis and Computer Science “A. Ruberti” (IASI), National Research Council (CNR), 00185 Rome, Italy

**Keywords:** senolytics, senomorphics, chronic diseases, aging, senotherapeutics, clinical trials, apoptosis, senescence

## Abstract

The WHO estimated around 41 million deaths worldwide each year for age-related non-communicable chronic diseases. Hence, developing strategies to control the accumulation of cell senescence in living organisms and the overall aging process is an urgently needed problem of social relevance. During aging, many biological processes are altered, which globally induce the dysfunction of the whole organism. Cell senescence is one of the causes of this modification. Nowadays, several drugs approved for anticancer therapy have been repurposed to treat senescence, and others are under scrutiny in vitro and in vivo to establish their senomorphic or senolytic properties. In some cases, this research led to a significant increase in cell survival or to a prolonged lifespan in animal models, at least. Senomorphics can act to interfere with a specific pathway in order to restore the appropriate cellular function, preserve viability, and to prolong the lifespan. On the other hand, senolytics induce apoptosis in senescent cells allowing the remaining non–senescent population to preserve or restore tissue function. A large number of research articles and reviews recently addressed this topic. Herein, we would like to focus attention on those chemical agents with senomorphic or senolytic properties that perspectively, according to literature, suggest a potential application as senotherapeutics for chronic diseases.

## 1. Introduction

Aging is a multifaceted process that includes the accumulation of senescent cells, loss of cell renewal at the organ level, and a significant organismal deterioration that increases the vulnerability to death [1]. Physiologically, several alterations accumulate progressively contributing to this picture, including (i) mitochondrial dysfunction; (ii) altered nutrient sensing; (iii) loss of proteostasis; (iv) alteration of intercellular communication; (v) exhaustion of stem cell reservoirs [1]. In this scenario, a series of molecular landmarks of aging has been associated with the cellular senescence in which cells undergo a state of permanent cell cycle arrest while the metabolism results active. In general, in a senescent cell, the reduction of telomerase activity and telomere length [2], an increase in DNA damage, paralleled by a reduction of DNA repair capacity, and a generalized DNA hypomethylation have been observed [1]. 

One of the features associated with the senescence process is protecting against cancer. In particular, p53 and p16 are critical regulators of the balance senescence/cancer, which are upregulated in senescent cells while down- or dysregulated in cancer [3]. The biological effect of the upregulation of these proteins is a permanent G1 arrest [3]. Recent comprehensive reviews, to which the reader is addressed, detail the molecular mechanisms of cellular senescence or its parallelism/antagonism with cancer [1,4,5].

Although the aging process appears unavoidable, the most recent evidence suggests that some intrinsic features of aging associated with the accumulation of senescent cells may become useful to design treatments aimed at preventing or reducing their damaging effect in a living organism [6].

During aging, physical, chemical, and biological agents, DNA replication errors, and reactive oxygen species continuously perpetuate damage to DNA and nuclear architecture [7]. As a result, somatic mutations accumulate, affecting gene transcription [8]. For instance, in senescent cells, an increase of progerin has been observed in association with telomere dysfunction [9]. The telomere length in senescence has been per se associated with reduced or absent telomerase activity [10,11,12]. In parallel, the whole chromatin structure undergoes changes involving global DNA hypomethylation or localized hypermethylation and histone post-transcriptional modifications (PTMs). During aging, specific PTMs may occur in the core histone N-terminal tails. They include the increase of H4K16 acetylation, H4K20 trimethylation, H3K4 trimethylation, and the decrease of H3K9 methylation and H3K27 trimethylation [13,14,15]. The presence of these modifications contributes to altered gene transcription, often ending up in a general increase in mRNA production [16]. 

Recently, it has been found that in old organisms, the cell-free mitochondrial DNA (cf-mt-DNA) accumulates, becoming a new potential marker of senescence [17]. The cf-mt-DNA can induce the activation of immune response worsening the inflammaging [17]. Here, inflammatory, mitochondrial, and lysosomal degradation pathways become frequently overrepresented as a transcription signature of aging in mammalian tissues [18].

A common feature of senescence is the accumulation of misfolded proteins intracellularly which enhances the formation of aggregates that might precipitate generating an interference with the physiological cellular functions and the presence into the extracellular matrix (ECM) of misfolded or not properly cleaved proteins that interfere with intercellular communication [19,20]. An example is given by the well-known aggregation of amyloid-beta or Tau peptides in Alzheimer’s disease (AD) [21]. The presence of misfolded proteins leads to increased secretory activity giving origin to the so-called senescence-associated secretory phenotype (SASP) [22]. The molecules secreted in the context of SASP are mostly cytokines, chemokines, and metalloproteases involved in inflammation and immunomodulation [22,23]. These molecules are at the basis of the chronic microinflammation often associated with chronic diseases in the elderly. Intriguingly, they may also be a potential therapeutic target to slow or diminishing the burden of aging. 

Besides, ECM has been found to play an active role in senescence [20]. In particular, during aging, the accumulation of mineralization, glycation, and the depletion of glycosaminoglycans on collagen reduces the sensitivity of ECM proteins to metalloproteinases resulting in inhibition of ECM remodeling [24]. In this condition, the mechanic integrity results strongly altered, accelerating deterioration of joints, skin wrinkling, heart fibrosis, and respiratory malfunctions. Moreover, in lung parenchyma and skin, the accumulation of racemization on aspartic acid residues reduces the turnover of elastin, another component of ECM [25,26]. The modification of elastin fibers reduces the elastic proprieties in the target organs or tissues, exacerbating emphysema in the lung, and atherosclerosis in arteries [27].

To further discuss the biological processes associated with senescence and aging is out of the scope of this article. For this, the readers are redirected to other more detailed articles [28]. Instead, we will focus here on the future potential application of the concepts illustrated below, which we believe useful to understand the development of future treatments of chronic diseases.

In recent years, various strategies have been attempted to prevent senescent cell formation and the inflammatory consequences of their accumulation. A natural compound named Resveratrol (RSV) has been demonstrated to have senescence-retardation properties have been demonstrated in animal [29,30,31]. RSV belongs to a class of polyphenols that can activate class III HDACs, the Sirtuins, protecting cells from oxidative stress, DNA damage, and senescence, realizing a so-called caloric restriction-like metabolic environment [32]. RSV is probably the prototype senomorphic compound with the demonstrated property of delay cellular senescence and prolonging life in invertebrates, fishes, and mice [30,31,32,33]. However, other in vivo experiments showed that RSV, lunularin, or dihydro resveratrol did not act as caloric restriction mimetics [34]. Due to controversial results, it is still unclear whether RSV may prolong life in non-human primates and humans [35,36,37]. Along a different line of research, instead of preventing senescence, Kirkland and coworkers, using repurposed anticancer drugs renamed as senolytics, attempted successfully to remove, at least partly, senescent cells from living organisms [38,39].

In particular, senomorphic molecules have been used to prevent or to slow down aging. Instead, the senolytics have been recently identified to induce apoptosis in senescent cells, reducing their number in specific organs.

Interestingly, other approaches have also been designed to prevent or revert the aging process. An example is the gene therapy intervention to preserve telomere function and DNA repair to prolong the organismal lifespan through the control of the expression of the telomerase reverse transcriptase (TERT) [40] or in haploinsufficient (Myc^+/−^) mice the accumulation of osteoporosis, cardiac fibrosis, and immunosenescence have been found decreased significantly [41]. 

Additionally, metabolically active substances that interfere with the production of SASPs, might envisage a critical non-pharmacological approach. An example are the glucocorticoids that suppress the secretion of interleukins, such as IL-6, IL-8, and that of cytokines or chemokines interfering with the IL-1α/ NF-κB pathway [42]. Along this line of research, it has been shown that the HMG-CoA reductase inhibitor Simvastatin by inhibiting prenylation in several proteins interferes with the onset of SAPS in senescent cells [43]. Additionally, the silencing of mTORC1 and PGC-1β causes a significant reduction in the total number of mitochondria slowing the cellular senescence through the reduction of reactive oxygen species (ROS) formation and the consequent DNA damage [44]. Despite the relevance of the genetic control of aging and the importance of reducing the production of inflammatory cytokines, these approaches are difficult or impossible to realize in humans or very inefficient on a body wide scale.

In the following paragraphs, we will describe the most critical characteristics, effects, and potential applications of selected members of senomorphic and senolytic molecules.

## 2. Senomorphics

The term “senomorphic” is referred to those molecules that delay or prevent senescence without affecting the total number of senescent or senescent-prone cells. In particular, senomorphic agents are aimed at controlling/reducing SASP, possibly prolonging the organismal lifespan. In this paragraph, we will review the state of the art of selected compounds with senomorphic properties tested in animal models or currently in human clinical trials.

The nordihydroguaiaretic acid (NDGA) increases the median lifespan by 8–10% in male mice but not in female, perhaps due to an unclear hormonal interference [45]. NDGA has been found to have anti-inflammatory properties that improve cellular metabolism [46]. NDGA’s primary target is the lipoxygenase that becomes inactive increasing fatty acid catabolism [46]. Additionally, NDGA upregulates the peroxisome proliferator-activated receptor α (PPARα) and the activated (phosphorylated) form of AMP-activated kinase resulting in better regulation of dyslipidemia and more efficient lipid metabolism [46]. The same study reports about the effect of Acarbose, an α-glucosidase inhibitor that inhibits the absorption of carbohydrates in the gut. In male mice, a positive effect has been observed with an average increase of lifespan of about 22%. In females, the same treatment exerted minimal effects [45]. The presence of 17-α-estradiol (EST) could determine this discrepancy, which, when present, is per se a protective factor against ischemic events and neurodegeneration [47]. In this context, EST alone administrated to males determined a 12% increase in the average lifespan [45]. 

Among senomorphics, Rapamycin inhibits the kinase mTOR [48], prolonged life of about 8% in rodent males, and 11% in females [48]. This observation suggested the presence of sexual dimorphism, which might be linked to the difference of genes expressed between males and females. It has been demonstrated that genes involved in the metabolism of xenobiotic chemicals and toxins (xenobiotic-metabolizing enzymes, XME) are more abundant in females than males. In particular, it has been shown that, upon Rapamycin treatment, transcription of XME genes is reduced in females, while it increases in males [48]. The reason for these conflicting results is unknown. However, it has been suggested that the sex-specific differences in the distribution of fat should be taken into account in future studies concerning Rapamycin and aging [48]. 

One critical factor correlated to the increase of lifespan is the fasting condition, which activates the group of histone deacetylases named Sirtuins. These molecules are not only able to modulate the structure of chromatin, allowing the activation of gene transcription, but participate in stimulating antioxidant and generally protective cellular mechanisms [49]. The overexpression of Sirt1, a member of the Sirtuin family, or its activation by RSV, induces autophagy. This effect has been demonstrated in human cells and in vivo in *C. elegans*, fishes, and rodents [50]. Remarkably, Sirtuins are among the targets of RSV, which, to a certain extent, mimics the protective consequences of fasting, at least in animal models. 

Recently, similar to RSV, the polyamine spermidine revealed anti-aging effects [51]. In particular, high levels of spermidine led to reduced acetylation of multiple lysine residues located at the N-terminal tail of histone H3. This effect was similar to that of RSV-activated sirtuins [51]. However, spermidine seems acting differently from RSV, exerting its effect by inhibiting histone acetylases instead of activating Sirtuins [52].

A recent study in humans suggested some anti-aging effects of Fluvastatin and Valsartan, two drugs used to treat cardiovascular diseases [53]. Specifically, the combination of Fluvastatin and Valsartan significantly increased the expression of Sirt1, telomerase activity, the 5’ AMP protein kinase catalytic subunit α 2 (PRKAA), and that of *KLOTHO* gene [53]. Coincidently, several intracellular protective pathways were activated [54,55]. 

To explore further the mechanisms involved in the protection from the consequences of aging, human skin fibroblasts from Hutchinson-Gilford progeria syndrome (HGPS) and Werner syndrome (WS) have been analyzed [56]. HGPS and WS are genetic diseases caused by the mutation of laminin A and DNA helicase genes, respectively [57,58]. KU-60019 is a compound that inhibits ataxia-telangiectasia-mutated (ATM) kinase, which is involved in the maintenance of mitochondrial function [59], and to restore the DNA integrity after a double-strand break [60]. In this study, the compound was administrated to senescent HGPS and WS fibroblasts. As a result, a significant reduction in the intracellular level of ROS and glycolysis has been observed [56]. In general, the effect of KU-60019 has been associated with the increase of mitochondrial membrane potential, determining a better metabolic function [56].

In conclusion, senomorphic molecules prevent or delay aging by negatively regulating pathways involved in inflammation, intracellular ROS production, fatty acids oxidation, DNA repair, and mitochondrial dysfunction. Table 1 summarizes the best-characterized senomorphic molecules which effect has been tested in an in vivo model.

Although senolytics are the most recently introduced and studied molecules in the field, conceptually opposed to them, other substances, protecting from apoptosis, are emerging as useful tools to contrast aging. In particular, the mitochondria-targeted antioxidants. Here we will give a concise mechanistic overview of these molecules. However, the readers are also invited to consider other more specialized and exhaustive articles on this topic [61,62,63].

In the cell, mitochondria are the primary source of ROS, which causes the alteration of mitochondrial membrane permeability transition (MPT), mitochondrial depolarization, swelling and cytochrome c (cyt c) release. In a neuronal cell line, it has been demonstrated that the treatment with the tetrapeptide named SS-31 (D-Arg-Dmt-Lys-Phe-NH2; Dmt=2’,6’-dimethyltyrosine) inhibits the ROS formation resulting in improved cell survival [64]. In aged brains, it has been observed a loss of activity of the mitochondrial nitric oxide (NO) synthase (mtNOS) associated with the reduction of mitochondrial complex IV [65]. As a result, in neuronal cells, the dysfunction of electron transport increases, enhancing the formation of ROS [66]. The addition of Vitamin E, acetylcarnitine, lipoic acid, and flavonoid-rich vegetable extracts have been observed to benefit in aging prevention by positively acting on mitochondrial function [67,68,69].

Mitochondrial alterations have often been observed in cardiovascular diseases (CVDs), particularly in the presence of hypertension. The administration of mitochondria-targeted superoxide dismutase mimetics inhibits the production of superoxide that relaxes the vascular endothelium with antihypertensive effects [70]. After an ischemia-reperfusion injury experiment, a recent study showed a positive effect of resveratrol, specifically on mitochondria [71]. Here, the treatment has been targeted to mitochondria by nanoparticles in order to inhibit apoptosis by reduction of ROS formation [71]. In vivo, the effect of this treatment also reduced the infarct area suggesting mitochondria-targeted antioxidants as a novel therapeutic intervention after cardiac injury [71].

In osteoarthritis, the cartilage degeneration is often a consequence of the action of inflammatory cytokines such as interleukin-1β (IL-1β) and tumor necrosis factor α (TNF-α) [72]. Upon the exposure on cytokines, the increase of NO production has found to cause damage to mitochondrial DNA (mt-DNA), triggering the apoptotic response [73]. To reverse this phenomenon, the gene encoding for the human DNA repair enzyme 8-oxoguanine DNA glycosylase/AP lyase (hOGG1) has been targeted to mitochondria reestablishing the integrity of mt-DNA and inhibiting the apoptotic pathway [74].

## 3. Senolytics

Senolytics are substances known to induce the death of senescent cells reducing their total number in vitro or in vivo. In a series of exciting experiments, to establish a model of aging-associated physical dysfunction, senescent adipose cells were transplanted from old to young mice, and molecules with senolytic properties were administered after the transplant [75]. In this condition, the combination of the antileukemic tyrosine kinase inhibitor Dasatinib and the antioxidant quercetin (D + Q) determined a significant reduction of senescence cells and the reduction of circulating pro-inflammatory cytokines [75]. As a result, the lifespan of treated mice was prolonged [75]. Notably, D + Q treatment was also beneficial in females in which the D + Q combination reduced age-related uterine disorders and fibrosis [76]. Moreover, D + Q has been tested in a clinical trial in which patients have been administrated for 3 days 100 mg of D and 1 g of Q orally [77]. As a results, after 11 days of the end of treatment, a general decrease of senescent cells by skin and adipose tissue biopsy have been observed [77].

Recent work reported the effect of magnetite nanoparticles coated with quercetin (MNPQs), targeted to senescent human fibroblasts [78]. This study reported that MNPQs were able to activate AMPK, which induced non-apoptotic death of senescence cells, and the reduction of pro-inflammatory response was reported [78]. Surprisingly, MNPQs improved mitochondrial oxidative phosphorylation and the glycolytic pathway suggesting a role as metabolic modulators [78].

Another compound that selectively kills senescent cells, is the EF24, a curcumin anolog [79,80]. Compared to curcumin, EF24 has been observed to be easiest to administrate and is more resistant to the degradation [79,80]. Its molecular mechanisms seem associated with the induction of BCL-2 family degradation via the proteasome pathway [81]. Although the EF24 senolytic proprieties have been well studied in cancer cells, little is known in healthy aging or in the presence of age-related chronic diseases [82]. Other natural product such as piperlongumine (PL), found in pepper fruit, has the property of modulating cellular lifespan. In particular, the administration of PL triggers apoptosis by the inhibition of the pro-survival Ras/PI3K/Akt/mTOR signaling axis and by the downregulation of surviving [83,84]. However, the PL role is well described in oncological studies such as in colon cancer, but further investigations are needed to clarify its effect on chronic diseases.

A different study reported a small molecule screening to identify novel substances with a senolytic effect [85]. Within this work, inhibitors of heat shock protein 90 (Hsp90) were identified as a novel class of senolytics [85]. These molecules can interact with the N-terminal of HSP90, involved in protein stabilization, degradation, homeostasis, and mitochondrial transport [86]. Specifically, Hsp90 inhibitors affecting the PI3K/AKT pathway, destabilizing AKT and inactivating their anti-apoptotic effectors such as NF-κB, mTOR, and FOXOA3 [86]. In mouse kidney, the effect of HSP90 inhibitors determined the reduction of the age-related marker p16^INK4A^ [85].

An example of an Hsp90 inhibitor is the compound 17-dimethylaminoethylamino-17-demethoxygeldanamycin, known as 17-DMAG, with the property to induce apoptosis in B-lineage acute lymphoblastic leukemia (B-ALL) cells [87]. In these cells, 17-DMAG elicited the expression of Hsp70, which inhibits cathepsin D and alters the autophagic flux resulting in cell death [88]. This finding suggests that Hsp90 inhibitors might have an impact on multiple pathways associated with cell survival and essential for senescent cells eradications. 

Navitoclax and its derivatives, anticancer therapeutics that bind BCL-xL, are a different category of inhibitors with senolytic properties [89]. An example is given by the compounds A-1155463 and A-1331852. They reduce the survival of senescent cells due to interference with the BCL-xL pathway [89]. Interestingly, these effects were evident in HUVEC and IMR90 cells, but not in preadipocytes [89]. The reason for the ineffectiveness on preadipocytes is still unclear, and little is known about the potential application of the compounds in chronic diseases [90,91]. Interesting in vitro experiments on senescent human renal epithelial cells, Navitoclax has been added in different concentration (from 0.004 to 1000 μM) [92]. In this study low dose (0.012 μM) reduces significantly the number of cells after 72 h of treatment confirming its role as senolytic molecule [92]. Additionally, Navitoclax effects have been tested in mouse model in which the rejuvenation of senescent bone marrow hematopoietic and muscle stem cells, the reduction of myeloid skewing and DNA damage have been observed [92].

Two antibiotics, Azithromycin and Roxithromycin, revealed senolytics properties promoting autophagy in senescent cells [93]. Notably, the Azithromycin not only induced autophagy but also increased the aerobic glycolysis [93] that is particularly active in senescent cells suggesting the potential beneficial effect of inducing cell death by accelerating metabolism in senescent cells [93].

The best-characterized senomorphic and senolytic molecules are summarized in Table 2.

## 4. Senotherapeutic Interventions in Models of Age-Related Diseases

### 4.1. Cardiovascular Diseases

In this section, we would like to summarize the potential application of senomorphics or senolytics in non-communicable chronic diseases that, in 2016, the World Health Organization (WHO) estimated to be the cause of about 41 million deaths yearly [94,95]. 

In the so-called Ink-Apoptosis Through Targeted Activation of Caspase 8 (INK-ATTAC) mice, the expression of FKBP-Casp8 has been induced under the control of *p16*^Ink4a^ promoter. This strategy is used to increase the expression of FKBP-Casp8 in those cells that have high levels of p16 [96]. In this model, the effect of a substance called AP20187, with the property of triggering apoptosis in senescent cells, was evaluated on physiological heart functions such as heart rate, ejection fraction, and ventricular thickness [96], parameters frequently altered in older adults [97]. Interestingly, in animals injected with AP20187, the cross-sectional area of ventricular cardiomyocytes (vCMs) differed substantially from treated and untreated animals. In particular, in the presence of AP20187, the diameter of vCMs was significantly smaller than in untreated mice [96]. Structurally, AP20187 is made by dimerization between mBAX (a recombinant protein derived from BAX, a pro-apoptotic factor) and FKBP (a neurotrophic molecule), which are proteins involved in the regulation of cellular death [98].

To investigate the effect of atherosclerosis on aging, ApoE^−/−^ mice have been fed with the western diet to facilitate the formation of atherosclerotic plaques [99]. Treated animals showed enrichment of nuclei positive for telomere-associated foci (TAF) in intimal aortic plaque compared to controls [99]. An independent group of ApoE^−/−^ mice received a weekly dose of Dasatinib and Quercetin (D + Q). This treatment determined the reduction of TAF positive cells due to a decrease in DNA damage [99]. Besides, the study revealed that D + Q administration increases the levels of phosphorylation in Serine 1177 of endothelial nitric oxide synthase (eNOS), suggesting for an improved nitric oxide signaling in atherosclerotic vessels with consequent reduction of plaque calcification and a generalized amelioration of the vasomotor dysfunction [99].

In a different study, small interfering RNAs (siRNAs) have been identified as novel senolytic molecules [100]. Six siRNAs emerged from a screening based on the property to trigger cell death in senescent preadipocyte [100]. In particular, those siRNAs interfered with the expression of ephrin (EFN) B1 and EFNB3, cyclin-dependent kinase inhibitor 1A (p21), plasminogen-activated inhibitor-2 (PAI-2), the phosphatidylinositol-4,5-bisphosphate 3-kinase delta catalytic subunit (PI3KCD), and BCL-xL, which globally are involved in viability and survival of senescent cells [100]. In the same study, to further investigate the senolytic effects in a mouse model of carotid dysfunction, D + Q has been administrated to partially knocked out ERCC mice (Ercc1^−/+^) to carotid function and reactivity was used to monitor the effects of the treatment. By this approach, an amelioration in the carotid response has been observed as early as five days after a single dose administration [100].

Navitoclax, a BCL-2 inhibitor, has been tested in a mouse model of myocardial infarction [101]. After treatment, the expression of p16 and p21 was reduced in CMs, and an improved ejection fraction was observed [101]. This finding suggests that Navitoclax might be considered an adjuvant treatment of patients after MI [101].

Cardiac Glycosides (CGs), such as digoxin, have been recently classified as novel senolytic drugs, active on senescent human fibroblasts [102]. CGs interfere with the Na^+^/K^+^ ATPase pump causing the depolarization and acidification of cytosol [102]. Senescent cells present a depolarized plasma membrane and a lower pH than younger cells resulting in an increase of sensitivity to the action of CGs, a phenomenon that drives cells into apoptosis [102].

In CMs isolated from a mouse model of heart failure (HF) caused by pressure overload, dipeptidyl peptidase 4 inhibitors (DPP4i) have been found playing a role in glucagon-like peptide 1 (GLP-1)/cAMP axis [103]. GLP-1 has been found as a substrate of DPP4, which cleaves and inactivates the GLP-1 N-terminal region, resulting in blocking the G protein activity [104]. After the administration of DPP4i in HF mice, the level of cAMP was restored, increasing cell survival [103]. As a general cardiac effect, it has been observed that DPP4i restored cardiac remodeling and contractile function, while CMs apoptosis is reverted, and circulating glucose level was decreased [103]. This finding suggests DPP4i as novel senomorphic drugs capable of preventing heart dysfunction under pressure overload. 

Another study demonstrated that in patients who undergo percutaneous coronary intervention (PCI), the administration of ABT-737, a senolytic molecule that acts as a BCL-2 inhibitor, induces platelets apoptosis [105]. Notably, in the platelets from low platelet reactivity (LPR) patients, the expression of BCL-2 is reduced compared to that of high platelet reactivity (HPR) patients [105]. The cause has been associated with an increased expression of miR-15b, a miRNA that targets Bcl-2 inducing apoptosis [105]. Interestingly, it has been observed that PCI patients with HPR are associated with an increased risk of ischemic/bleeding events suggesting the modulation of miR-15b or the administration of ABT-737 as a novel treatment to reduce risks in HPR patients after PCI [105].

In a mouse model of heart transplantation, the D + Q combination has been administrated to verify whether the clearance of senescent cells, an important source of pro-inflammatory cf-mt-DNA, improves the inflammatory response and ameliorates the risk of adverse outcome after receiving old organs [17]. As a result, the treatment of donor mice with D + Q prolonged the survival of allograft, suggesting the clinical potential of senolytics, possibly enabling the clinical use of organs from older donors [17]. 

### 4.2. Respiratory Diseases

The D + Q combination has also been tested in patients with idiopathic pulmonary fibrosis (IPF), a chronic condition in which abundant fibrotic tissue forms between the alveoli interfering with the gas exchanges, by oral administration three days/week for three consecutive weeks [106]. Some physical and pulmonary parameters such as the six-min-walk distance (6MWD), the four meters usual gait speed, the timed five-repetitions chair-stands, the short physical performance battery (SPPB), the grip strength, the forced vital capacity (FVC), and the forced expiratory volume in 1-s (FEV1) have been evaluated [106]. In this experiment, the 6MWD, walk speed, and the SPPB gained more than 5%, while pulmonary functions did not change. Possibly, the D + Q removes senescent cells but does not affect the ECM already synthesized [106]. 

IPF is known as associated with cellular senescence. The demonstration derives from the observation of sections of human lung tissue obtained from IPF patients where many positive cells for p16, TAFs, and DNA foci have been found [107]. To further explore the effect of D + Q, the combination was tested on human fetal lung fibroblast (IMR90), and it has been observed that the administration of D + Q increased the clearance of senescent cells [107]. In these experiments, INK-ATTAC mice with IPF were used to investigate the efficacy of D + Q further. In these animals, senescence was induced through aerosolized intratracheal bleomycin; successively, mice were treated with AP20187 or D + Q [107]. As a result, treated mice showed clearance of senescent cells, loss of weight, reduction of fibrosis, and reduction of pro-inflammatory cytokines production [107]. Notably, these mice were treated in the early stages of IPF, while in humans, the condition is usually treated in later stages; this discrepancy might be a limiting factor in designing interventions with senolytic agents in IPF and requires much more investigation to understand whether a senolytic approach could be useful in this human pathophysiological context.

In end-stage pulmonary disease, a severe condition in which lung transplantation is the sole therapeutic option currently available, the effects of DPP4 inhibition have been studied in rats [108]. In animals, the tissue damage was induced by ischemia/reperfusion (IR) before pulmonary transplantation [108]. Before surgery, the bis(4-acetamidophenyl)-1-(*S*)-prolylpyrrolidine-2-(*R*, *S*)-phosphonate, alias AB192, was administered to inhibit the activity of DPP4 [108]. As a result, oxygenation was preserved, free oxygen radicals, pulmonary edema, and neutrophil sequestration decreased, suggesting that AB192 might help preserve lung function. This evidence suggests that DPP4 inhibition could be considered a preventive treatment before lung transplantation in humans [108].

Chronic asthma is another pathological context in which DPP4 might be involved. In bronchial epithelial cells of asthmatic patients, DPP4 levels increase in response to IL-13 [109]. Interestingly, a correlation emerged between DPP4 and the expression of the inducible nitric oxide synthase (iNOS) responsible for oxidative damage [109]. However, further studies are needed in order to understand the role of DPP4 in the pathophysiology of human asthma. 

Remarkably, increasing DPP4 expression has been observed in lung adenocarcinoma, a type of cancer frequent in the elderly [110]. Here, in vivo mouse models, DPP4 inhibition was obtained by the administration of Vildagliptin, a substance that is an inhibitor of DPP4 activity commonly used in treating diabetes mellitus type 2 [110]. The first result, observed in treated mice, showed the increase of macrophages and natural killer cells (NK). In contrast, in vitro, the administration of Vildagliptin to lung cancer cells allows the production of surfactant proteins which are related to an inflammatory condition [96]. In particular, in tumor cells derived from Vildagliptin treated mice, it has been noticed that NK cells expressed tumor necrosis-related apoptosis-inducing ligand (TRAIL), which induced the expression of γH2AX, which is known to be a marker of stress [110]. This study suggests that lung cancer growth could be arrested by Vidalgliptin through the TRAIL-cytotoxicity pathway, highlighting the senolytic and beneficial effects of this compound [110].

In chronic obstructive pulmonary disease (COPD), the level of metalloproteinases-9 (MMP9) might increase in consequence of Sirt1 reduction [111]. In the sputum of COPD patients, the addition of SRT2172, a selective Sirt1 activator, inhibited the expression of MMP9 [95]. In the COPD mouse model, the exposure to the smoke of cigarettes enhanced the MMP9 transcription in the lungs and globally increased the number of neutrophils and macrophages [111]. In this experiment, SRT2172 has been administered intranasally, resulting in better tolerance to exercise and oxygenation ameliorating the lifespan of animals [111].

Also, Navitoclax has been used to study pulmonary emphysema, a condition that can be caused by cigarette smoke [112]. In mice exposed to smoke, the preventive administration of Navitoclax removes p19^ARF^ expressing cells from the lung resulting in an improvement of pulmonary functions, such as the pressure-volume loop and a reduction of inflammation [112].

A natural compound, the sulforaphane, which derives from *cruciferaceae*, is known for its detoxification of xenobiotics properties. In the lung, the compound has been found to inhibit IL-8 production, reducing the inflammation caused by SASPs upon pollutant stimulation [113]. In particular, in a mouse model of acute lung injury, the sulforaphane acted as a pulmonary protector, realizing a connection between mitochondrial metabolism and the NF-κB pathway [114], modulating the activation of the NF-E2-related factor 2 (Nrf2) that regulates the antioxidant pathway [114]. In these mice, the sulforaphane was administrated orally and determined a decrease in lung injury [114]. However, the senolytic role of sulforaphane has been shown only in glioblastoma stem cells in which induced apoptosis, triggering ROS formation [115]. Further studies on non-tumor associated respiratory diseases are urgently needed to understand whether this natural compound could be used in human clinical protocols.

In a rat model of acute pulmonary thromboembolism (PTE), the properties of RSV have also been explored [116]. Here, in vitro experiments were performed on isolated rat pulmonary artery endothelial cells, where the treatment with RSV revealed its ability to interfere with the p38MAPK pathway, reducing the production of monocyte chemoattractant protein-1 (MCP1) as well as the presence of inflammatory infiltration [116].

### 4.3. Neurocognitive Diseases

In the brain, Tau protein aggregation and precipitation increase cellular senescence contributing to the exacerbation of neurodegenerative diseases such as Alzheimer’s disease (AD) [117]. Interestingly, transgenic mice in which Tau has been overexpressed, the treatment with D + Q reduced the formation of neurofibrillary tangles, determining an increase in senescent apoptotic cells and a better cerebral blood flow [117].

AD is characterized by the accumulation of TAU protein [118] and β-amyloid plaques, which might exacerbate a depression-like behavior [118]. The administration of SB203580, a molecule that inhibits p38MAPK, has been observed to ameliorate the depression [119]. However, in rat models, SB203580 has been injected intra-peritoneally, making this type of administration still not available in clinical treatment [119].

In AD patients and AD mouse models, it has been observed that oligodendrocyte progenitor cells (OPCs) exhibit a senescent phenotype in the presence of amyloid-beta plaques [120]. In this condition, OPCs upregulated the expression of p16 and p21 genes and became SA-β-Gal positive, acquiring a senescent pro-inflammatory phenotype [120]. These alterations did not appear in other cell types such as microglia, astrocytes, and oligodendrocytes, which were not different from those of non-AD patients or normal control mice [120]. In this context, D + Q treatment caused a significant reduction in senescent cells [120]. As a result, the formation of amyloid plaques was significantly reduced, and the learning memory improved in association with the clearance of hippocampal senescent OPCs [120]. Inhibitors of DPP4 have also been tested in AD to restore the hippocampus functionality [120]. In this context, the inhibition of DPP4 improved the stabilization of the SDF-1α/CXCR4 axis, incrementing circulating stem cell progenitors into the damaged brain region [121].

Another age-related neurological disease is Parkinson’s disease (PD), in which dysfunction of dopaminergic (DA) neurons is one of the predominant features. A recent study revealed that a DNA binding protein, named Special AT-rich sequence-binding protein 1 homeobox 1 (SATB1), is required to repress the cyclin-dependent kinase inhibitor 1A (CDKN1A) in order to preserve the microglia activation [122]. In PD patients, the STAB1 expression was decreased in association with an early onset of cellular senescence [122]. In vitro experiments with SATB1 knockdown cells, indicating an increase of p21 expression, SASP production, mitochondrial dysfunction, and protein oxidation, suggesting the SATB1 could be a potential target for a new PD treatment [122]. Interestingly, in an animal model of PD realized in rats, it has been demonstrated that the administration of quercetin improves catalepsy suggesting for a new potential treatment to restore the physiological dopaminergic signal [123]. Neurocognitive disorders are also common in patients with brain metastasis who undergo irradiation therapy [124]. A study demonstrates that, in this condition, the administration of navitoclax improves the cognitive functions by depleting senescent astrocytes [124]. This finding copes with the concept of senolytic molecules used in cancer therapy and neurocognitive treatment during aging.

### 4.4. Type 2 Diabetes

In recent years, in low and middle-income countries, diabetes mellitus type 2 (T2D) has risen dramatically; indeed, about 422 million people worldwide are currently affected, with 1.6 million deaths reported each year [125]. T2D is often associated with obesity, accelerated aging, and inflammation [126]. In obese people, senescent cells accumulate in the visceral fat [127]. Interestingly, in the 3MR and INK-ATTAC mouse models, the administration of Ganciclovir or AP20187 or D + Q promoted clearance of senescent cells, improving the glucose tolerance [126]. Moreover, adipogenesis was promoted, suggesting that the elimination of senescent cells can be replaced by non-senescent and proliferating adipocyte progenitors that can then differentiate into insulin-responsive fat cells [126]. Regarding inflammation, it decreased after senolytics administration; in this condition, a reduced macrophage homing has been found compared to untreated mice [126], suggesting that senotherapeutic interventions might alleviate metabolic dysfunction.

Notably, accelerated aging of pancreatic islets, especially the β cell component, determines diabetes [127]. In the INK-ATTAC mouse model, the expression of SA-β-Gal, p16, SASPs, and inflammatory cytokines, such as CCL4 and IL6, is increased compared to normal mice [127]. The administration of Navitoclax determined a generalized functional improvement [127] associated with a significant decrease in the blood glucose level [127]. Consistently, in human samples of pancreatic islets obtained from T2D patients and healthy donors, a significant accumulation of the age-related biological marker was found in the T2D samples [127].

Interestingly, senescent β cells upregulated transcription of the anti-apoptotic molecules BCL-2, BCL-xL, BCL-w, resulting in the activation of a pro-survival pathway [128]. In this condition, a BH3-mimetic has been used to trigger apoptosis in senescent cells isolated from the islets of a T1D mouse model, reporting a beneficial effect on blood glycemia [128]. This effect has not been seen with other BCL-3-selective inhibitors such as Venetoclax or ABT-199. However, these compounds were still able to reduce senescent cells and inflammation [128].

In T2D, cardiovascular complications are typical findings [129]. In a study conducted on a diabetic rat model, Simvastatin, an HMG-CoA reductase inhibitor, reduced cardiac hypertrophy and hyperglycemia, preventing the deposition of collagen and fibrosis [130]. In CMs upon Simvastatin administration, the expression of NF-*κ*B decreased with a global reduction of inflammation [130]. However, the senomodulator effect of Simvastatin has been demonstrated in breast cancer in which the drug inhibited the prenylation of proteins suppressing SASPs [43] and slowing tumor progression [43]. Despite the positive effect of Simvastatin in breast cancer, further studies are needed to understand whether this molecule has a role as a real senotherapeutic molecule in aging-related diseases.

### 4.5. About Ongoing Clinical Trials

The number of ongoing clinical trials in which synthesized small molecules or natural compounds have been used to induce senolysis in humans is growing, but the number of subjects involved is limited, and only a few results have been made yet available. The following is a summary of recent or ongoing clinical trials published on ClinicalTrials.gov focused on senolytics and their effect on older people with chronic pathological conditions.

Since May 2020, the effect of senolytic drugs is going to be tested in order to improve skeletal health in candidates more than 70 years old (ClinicalTrial identifier: NCT04313634). In detail, two groups of participants are going to be investigated: one group is treated with D + Q (100 mg for two days consecutively and 250 mg for three days consecutively respectively), the other group will receive only fisetin (100 mg for two days consecutively) (ClinicalTrial identifier NCT04313634). These drugs will be administered orally. The primary purpose of this trial is the validation of senolytic molecules as supportive care (ClinicalTrial identifier NCT04313634).

In a different study, the effect of the combination D + Q is under evaluation during the early onset of AD (ClinicalTrial identifier NCT04063124). This study aims to investigate whether the D + Q combination can penetrate the blood-brain barrier and whether this combination may ameliorate AD symptoms (ClinicalTrial identifier NCT04063124).

A further study evaluates the effect of D + Q on people affected by diabetic chronic kidney disease with age ranging from 40 to 80 years (ClinicalTrial identifier NCT02848131). The study aims to evaluate the D + Q effect on frailty and inflammation not only in older people but also in those who show signs of early onset of the disease (ClinicalTrial identifier NCT02848131).

A new molecule, named UBX0101, is under the investigation of scientists to evaluate its effect on the degeneration of articular cartilage in knees (knee osteoarthritis) that is common in the elderly. In the long-term follow-up (12 weeks) of patients with painful knee osteoarthritis, the administration of UBX0101 by a single intra articular injection at the doses 0.5 mg, 2.0 mg, or 4.0 mg did not show any reduction of pain compared to placebo (ClinicalTrial identifier NCT04349956). In light of this finding, another Clinical Trial (ClinicalTrial identifier NCT04229225) is considering UBX0101 as a potential therapy for knee osteoarthritis (ClinicalTrial identifier NCT04229225). In this study, two doses, higher than those used in the ClinicalTrial NCT04349956, will be tested, 4 and 8 mg, in a single dose by intraarticular injection. The pain of each patient will be evaluated for pain reduction after 12 and 24 weeks of UBX0101 administration (ClinicalTrial identifier NCT04229225).

Fisetin is also under scrutiny in a study aimed to attenuate the consequences of osteoarthritis in knees (ClinicalTrial identifier NCT04210986). 20 mg/kg of Fisetin will be administrated orally for two consecutive days, followed by 28 days off, then two more consecutive days of fisetin. Patients will be evaluated for SASPs and other inflammatory biomarkers integrated with magnetic resonance and physical performance (ClinicalTrial identifier NCT04210986). The end-points of this trial will be the dose-finding of fisetin, the overall duration of the treatment, the improvement of joint health, and the reduction of osteoarthritis symptoms (ClinicalTrial identifier NCT04210986)

Interestingly, in older adults, the markers of frailty, inflammation, insulin resistance, and bone resorption and bone formation have been evaluated after the administration of Fisetin (ClinicalTrial identifier NCT03675724). This clinical trial aims to find new biological markers of inflammation and frailty that no one has evaluated yet. The oral administration of Fisetin amounted to 20 mg/kg/day for two consecutive days (ClinicalTrial identifier NCT03675724).

Also, Rapamycin, a molecule that acts as senomorphic [48], will be tested in different doses in order to establish a long-term safety profile by the evaluation of biochemical and physiological parameters such as change in visceral fat mass, bone density, and liver functions in healthy older adults. Specifically, 1.5 mg/day for three days per week; 2.5 mg/day for three days per week; 5 mg/day once per week; 5 mg/day twice per week will be administered (ClinicalTrial identifier NCT04488601).

## 6. Conclusions

**Figure 1:**
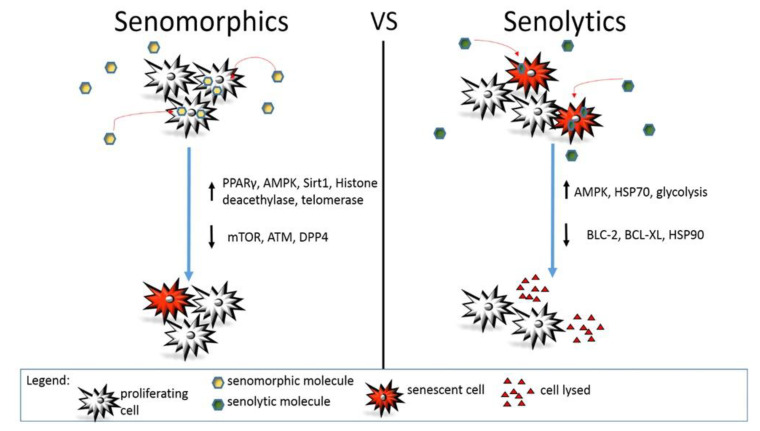
Action mechanism of senomorphic vs. senolytic compounds. Different molecular pathways are modulated by senomorphic or senolytic drugs. In general, senomorphic molecules delay or prevent senescence without affecting the total number of senescent or senescence-prone cells. On the contrary, senolytics induce death in senescent cells reducing their total number in the body or a specific organ.

Aging is a slow and unavoidable process during which the organism progressively loses the capability of cell renewal, and the function of many organs results in altered cellular pathways [1,2,11,40,43,128]. Many small molecules, called senolytics, can reduce senescent cells by promoting apoptosis. They act on specific pathways relevant to senescent cell survival (see Figure 1). Their modulation and the consequent reduction in the number of aged cells often determine a general functional improvement [61,62,70,80,84,91,92,108,110]. In this review, the authors summarized recent information about the effect of senomorphics, senolytics, and other even more recent anti-aging strategies in the context of the most common age-related chronic diseases. Special attention has been paid to overviewing the most relevant ongoing clinical trials in the field. Given the constant progression of the average population age worldwide and particularly in western countries, and the parallel increase in chronic disease occurrence, new treatments are urgently needed. Based on the current evidence, it is conceivable that new advances will come out from the lines of research considered in this article that might soon provide better innovative therapeutic solutions for chronic diseases.

Table 3 summarizes some of the information about senolytics and their clinical perspectives.

## Figures and Tables

**Table 1:** Selected senomorphic drugs and their effects.

Senomorphic	Target Pathway	Effects	Reference
NDGA	Upregulation of PPARγ	Regulation of dyslipidemia	[45,46]
Acarbose	Upregulation of PPARγ	Increase of lifespan	[45]
Estradiol	Upregulation of PPARγ	Increase of lifespan	[45]
Rapamycin	mTOR is inhibited	Increase of lifespan	[48]
Sirt1	Upregulation of AMPK	Increase of fatty acid oxidation and improvement of mitochondrial functions	[49]
RSV	Sirt1	Amelioration of oxidative stress	[50]
Spermidine	Histone deacetylase	Increase of lifespan	[51,52]
Fluvastatin and Valsartan	Upregulation of Sirt1, PRKAA, telomerase, and KLOTHO	Amelioration of glucose and fatty acid oxidation	[53,54]
KU-60019	Inhibition of ATM	Improvement of mitochondrial function	[56]

**Table 2:** Selected senolytic molecules and their effects.

Senolytic Molecule	Target Pathway	Effects	Reference
D + Q	Upregulation of AMPK	Reduction of senescent adipocyte and senescent skin cells	[77]
Quercetin	Upregulation of AMPK	Reduction of inflammation and senescent cell death	[75]
EF24	Proteasome degradation of BLC-2 family members	Apoptosis in senescent cells	[79,81]
Hsp90 inhibitors	Alteration of the PI3K/AKT pathway	Activation of a pro-apoptotic pathway in senescent cells	[85]
17-DMAG	Upregulation of HSP70	Increase of autophagic flux	[88]
A-1155463 and A-1331852	Inhibition of BCL-XL pathway	Lysis of senescent cells in specific cell lines	[89]
Azithromycin and Roxithromycin	Enhancement of aerobic glycolysis	Induction of senescent cell death	[93]

**Table 3:** The effect of senolytics in age-related chronic disease. Abbreviations: HPR: high platelet reactivity; T2D: diabetes mellitus type2; T1D: diabetes mellitus type1; AD: Alzheimer’s disease; PD: Parkinson’s disease; IPF: idiopathic pulmonary fibrosis; COPD: chronic obstructive pulmonary disease; PTE: pulmonary thromboembolism; D + Q: Dasatinib and quercetin.

DRUG	DISEASE	EFFECT	REFERENCE
ATB-737	HPR	Might reduce the risk of ischemic and bleeding events	[105]
	T2D	Amelioration of Ca^2+^ signaling in vessel cells	[129]
AP20187	cardiovascular	Cardiac fibrosis and myocardial hypertrophy are reduced	[96]
	T2D	Improvement of glucose tolerance. Increase of hepatic glucagon and muscular glucose uptake	[98]
D + Q	atherosclerosis	DNA damage is reduced, and improvement of vasoconstriction	[99]
	IPF	Amelioration of walk speed and resistance	[106]
	AD	Improvement of learning and memory	[120]
	T2D	Increase of adipogenesis	[125]
	Skeletal health	ONGOING CLINICAL TRIAL	(ClinicalTrial identifier: NCT04313634)
	AD	ONGOING CLINICAL TRIAL (D + Q cerebrospinal diffusion)	ClinicalTrial identifier NCT04063124
	Diabetic chronic kidney disease	ONGOING CLINICAL TRIAL	ClinicalTrial identifier NCT02848131
Digoxin	Na^+^/K^+^ATPase pump disbalance	Regulation of cellular pH	[102]
DPP4 inhibitors	Heart failure	Amelioration of heart functions	[104]
	Pulmonary disease	Amelioration of oxygenation and reduction of edema	[108]
	Lung adenocarcinoma	Block of lung cancer growth	[110]
Fisetin	Knee osteoarthritis	ONGOING CLINICAL TRIAL	ClinicalTrial identifier NCT03675724
	Frail Elderly syndrome	ONGOING CLINICAL TRIAL	ClinicalTrial identifier NCT04210986
Quercetin	PD	Amelioration of catalepsy	[123]
Rapamycin	Reducing clinical aging measures	ONGOING CLINICAL TRIAL	ClinicalTrial identifier NCT04488601
RSV	PTE	Reduction of inflammation	[116]
Navitoclax	Pulmonary emphysema	Improvement of pressure-volume loop and reduction of inflammation	[124]
	Brain metastasis	Cognitive performance is increased	[124]
SB203580	AD	Improvement of memory deficit	[119]
SRT2172	COPD	Sirt1 activity is increased, and the oxygenation is improved	[111]
Sulforaphane	Lung injury	The inflammation and SASPs are reduced	[114]
UBX0101	Knee osteoarthritis	FAILED CLINICAL TRIAL	ClinicalTrial identifier NCT04349956
	Knee osteoarthritis	ONGOING CLINICAL TRIAL	ClinicalTrial identifier NCT04229225
Venetoclax	T1D	The production of pro-inflammation cytokines is decreased	[128]
